# Statistical modeling of Si-based refractory compounds of bamboo leaf and alumina reinforced Al–Si–Mg alloy hybrid composites

**DOI:** 10.1038/s41598-023-31364-7

**Published:** 2023-04-03

**Authors:** Olanrewaju S. Adesina, Adeolu A. Adediran, Francis O. Edoziuno, Olufemi O. Sanyaolu, Babatunde A. Obadele

**Affiliations:** 1 Department of Mechanical Engineering, Redeemer’s University, P.M.B. 230, Ede, Osun State Nigeria; 2 Department of Mechanical Engineering, Landmark University, P.M.B. 1001, Omu - Aran, Kwara State Nigeria; 3 Department of Metallurgical Engineering, Delta State Polytechnic, Ogwashi-Ukwu, 320107 Nigeria; 4 Department of Chemical, Materials and Metallurgical Engineering, Botswana International University of Science and Technology, Palapye, Botswana

**Keywords:** Engineering, Materials science

## Abstract

Wear properties of Al–Mg–Si alloy matrix hybrid composites made with Si-based refractory compounds (SBRC) derived from bamboo leaf ash (BLA) as complimentary reinforcement with alumina have been studied. The experimental result indicate that optimum wear loss was obtained at higher sliding speed. The wear rate of the composites increased with an increase in BLA wt. %, with the composites having 4%SBRC from BLA + 6% alumina (B4) showing the least wear loss for the different sliding speeds and wear loads considered. With increasing BLA weight percent, the composites' wear mechanism was mostly abrasive wear. Numerical optimization results using central composite design (CCD) reveal that at a wear load of 587.014N, sliding speed of 310.053 rpm and B4 hybrid filler composition level respectively, minimum responses in wear rate (0.572mm^2^/min), specific wear rate (0.212cm^2^/g.cm^3^) and wear loss (0.120 g) would be obtained for the developed AA6063 based hybrid composite. Perturbation plots indicate that the sliding speed have more impact on wear loss, while wear load have significant impact on the wear rate and specific wear rate.

## Introduction

Manufacturers are constantly in search for materials having a good strength to weight ratios for automobile and aerospace application. To this, composites materials especially aluminium metal matrix composites (AMMCs) reinforced with synthetic materials such as SiC, Al_2_O_3_ etc. are highly sought after^1^. Hybrid composites have been widely used by researchers to achieve an improved wear resistance when particulate reinforcement of Al_2_O_3_ was used in the design of AMMCs^2^. From their work authors^3^, reported that weight percent (wt.%) of reinforcement is the most significant factor influencing the wear rate of the composites they developed. They noted that, load and sliding speed followed concurrently in the ranking order of significant. Findings from^4^ concluded that the hybrid composites developed exhibit an excellent wear resistance compared to the base alloy and monolithic composite respectively. The statistical results obtained from the orthogonal array using Taguchi for sliding speed, applied load, sliding distance and wear rate further corroborate the results.

In previous studies, rice husks (RHs) were successfully utilized in the production of Si-based refractory compounds (SBRC)^5^. These refractory compounds were reported to contain SiC polytypes which can serve as reinforcement materials in the design of AMMCs^6^. The investigation on the comparison of wear and friction behaviour of aluminium matrix alloy (Al 7075) and silicon carbide based aluminium metal matrix composite under dry condition at different sliding distance was reported^7^. They showed from their results that the wear rate of AMMCs reinforced with SiC was about 25–45%. On the other hand, about 14% improvement in wear resistance was observed when SiC was used as reinforcement in the design of AMMC as against monolithic alloy^8^.

In this study, we attempt to use the SBRC derived from BLA as complimentary reinforcement with alumina using central composite design (CCD) to statistically analyse, model and optimize the experimental results. The findings from this study would contribute to the existing data base on the optimization of selected wear parameters of Si-based refractory compounds derived from bamboo leaf and alumina reinforced Aluminium 6063 (AA6063) alloy.

## Materials and methods

### Materials

AA 6063 (Al–Mg-Si) is the base material used for this study, which was supplied as an ingot by a local vendor. The elemental composition was obtained using a spark spectrometer and the result is shown in Table 1. The reinforcement materials used in this study are alumina with a particle size range of 30 µm, and Si-based refractory compounds (SBRC) derived from bamboo leaf with particle size < 60 µm. Magnesium was used to enhance the wettability between the base material and the reinforcements. Details about the synthesis route for the development of the SBRC were previously reported by^5^.

**Table 1:** Elemental composition of Al–Mg-Si alloy.

Element	Wt.%
Fe	0.2001
Si	0.4300
Cu	0.0100
Zn	0.0130
Ti	0.0200
Mn	0.0120
Mg	0.4800
Pb	0.0010
Cr	0.0101
V	0.0020
Ni	0.0110
Ca	0.0011
Cd	0.0001
Na	0.0002
Al	98.8094

### Composites production

Double stir casting was used to produce the composites following the protocol reported by^9^. Before casting, charge calculations were done to determine the mass of alumina and SBRC of bamboo leaf ash (BLA) required to produce a 10 wt.% reinforcement. Table 2 shows the mix ratio of the reinforcements and their designation.

**Table 2:** Sample designation and weight ratio of the reinforcements.

Sample designation	Bamboo leaf ash (BLA)	Al_2_O_3_
C1	0	10
B2	2	8
B3	3	7
B4	4	6
C2	10	0

The SBRC of BLA and alumina (Al_2_O_3_) particles were preheated at a temperature range of 200–300 °C in other to remove the moisture and aid the wettability with the base alloy melt. The Aluminium 6063 ingots were introduced into a crucible furnace operated at 760 ± 30 °C and the melt was allowed to cool in a semi-solid state in the furnace at about 600 ± 20 °C. A temperature probe was used to regulate the furnace temperature. At the temperature earlier stated, preheated SBRC of BLA, Al_2_O_3,_ and 0.15 wt. % magnesium powders were added and stirred manually for 10–15 min. The composite slurry was then superheated to 790 ± 30 °C and a second stirring was achieved through a mechanical stirrer. The stirring process was obtained using 350 rpm motorized stirrer for 15 min to aid the distribution of the particles in the molten Al–Mg-Si alloy. The liquefied composites were then cast into ready sand moulds with chills inserted as reported by^9,10^.

### Wear test

The wear test of the composites was achieved using a Taber Abrasion wear testing machine (TSE-A016) following ASTM G195-18 standard^11^. The wear test required mounting disc-shaped arranged samples having 200 mm diameter and 5 mm thickness on the turntable platform of the wear machine. The samples were rapt at a constant pressure by two abrasive wheels lowered onto the sample surface. The turntable rotates with the samples which drive the abrasive wheels in contact with its surface. The rubbing action between the sample and the abrasive wheel generates loose composite wear debris as the rotating motion continues. The test was piloted for 15 min, and the weights of the samples were recorded before and after the tests. The Taber Wear Index was thereafter evaluated using the relation^12^

1 $${\text{Wear}}\;{\text{index = }} \frac{Initial\;weight - Final\;weight}{{Time\;of\;test\;cycle}} \cdot 1000$$

where the initial and final weights are in grams, and the time of the test cycle in minutes.

### Statistical analysis, modeling, and optimization of wear parameters

Central composite design (CCD) type of the response surface methodology (RSM) experimental design of the Design Expert Software file version 11.1.2.0 was used to statistically analyses, model and optimize the experimental results. A reduced cubic design model was applied during the analysis. The considered wear process parameters (speed & load) were set as the independent variables (numeric factors A & B) and the hybrid filler weight ratios (%wt) as the categoric factor (C) with five levels (C_1_, B_2_, B_3_, B_4_ & C_2_). Wear rate (mm^2^/min), specific wear rate (cm^2^/g.cm^3^), and mass loss (g) were set as the response variables (Responses 1 to 3). Forty-five (45) experimental runs were performed to obtain the responses of the dependent variables/composite wear properties. Statistical significance of the experimental results was evaluated using ANOVA. Diagnostic plots and model graphs were used to analyze the developed statistical models. Numerical optimization solution shows the optimal level of the composite and wear parameters/factors that will result in minimum wear rate.

### Ethics approval

Collection of plant material, comply with relevant institutional, national, and international guidelines and legislation.

## Results and discussion

### Wear loss

**Figure 1:**
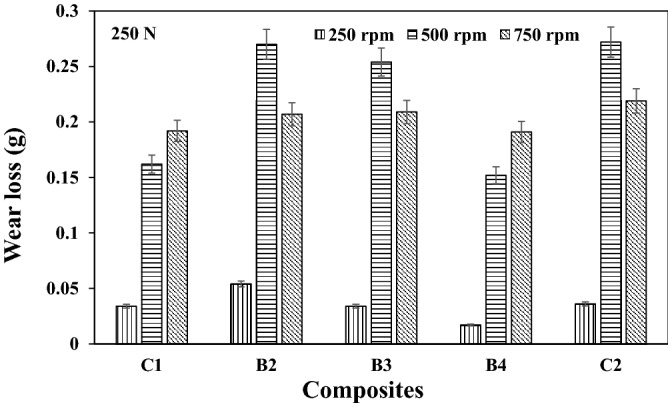
Wear loss at load 250 N under different speed for the composites.

**Figure 2:**
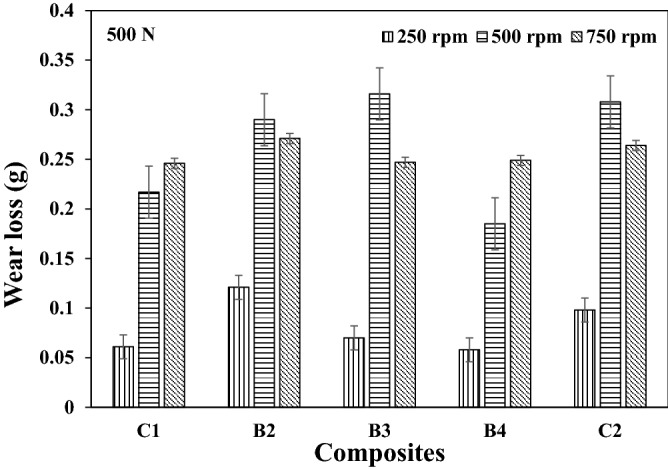
Wear loss at load 500 N under different speed for the composites.

**Figure 3:**
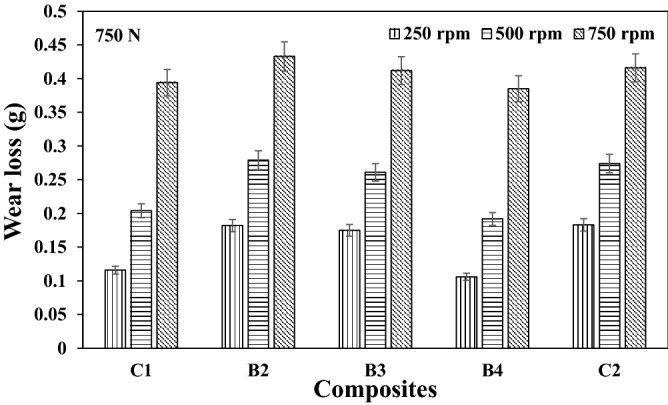
Wear loss at load 750 N under different speed for the composites.

Representative plots showing the variations in wear loss for the different speeds used in this study are as presented in Figs. 1, 2, and 3 respectively. It is evident from Figs. 1 and 2 that an optimum wear loss were recorded at higher speed. The rate of material removal was observed to be high as the speed increases, thus increasing the wear loss. Sample B4 had the least wear loss for the different speed used. The lesser the material removal rate was as a result of the resistance to microcutting by the composite owing to the reinforcement history. It is further noted in Fig. 3 that there is a transition domain from mild to severe wear as compared to the trend in Figs. 1 and 2 respectively (lower loads). Furthermore, the transition from mild to severe wear shifts to higher wear loss at constant load of 750 N under varying speeds. The sliding wear regime could be classified into three categories which are ultra-mild, mild and severe wear. As load increases with increasing sliding speed, it is common to observe an increase in the wear rates/loss. Most time, severe wear rate/loss is about three orders magnitude higher than mild wear. From Fig. 3, it could be observed that wear loss increased by about three other magnitude from sliding speed of 250 rpm to 750 rpm. It is also noted that at higher load application, the variation in the wear loss was within the range of 30–75%. The reduction in the wear loss was as a result of the oxide phases in the reinforcement materials. This observation was corroborated by the findings from Idusuyi & Olayinka^13^.

### Wear rate

**Figure 4:**
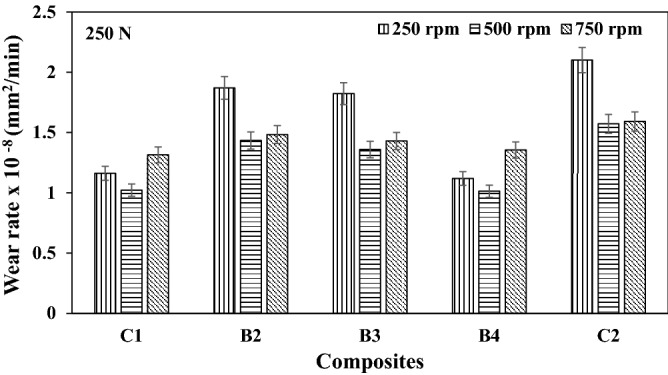
Wear rate of composites at load 250 N under different speed.

**Figure 5:**
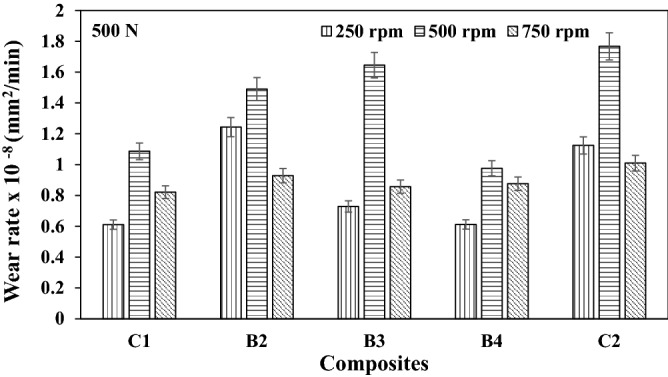
Wear rate of composites at load 500 N under different speed.

**Figure 6:**
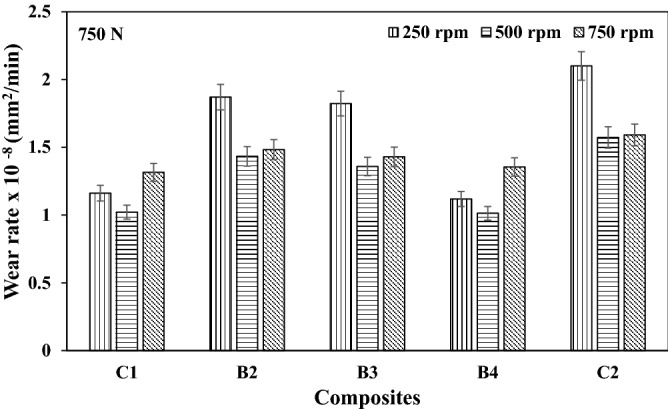
Wear rate of composites at load 750 N under different speed.

The variation in the wear rate of the composites are as presented in Figs. 4, 5, and 6 respectively for different speeds. It can be seen from this result that the reinforcement materials tend to restrict plastic deformation, hence forming a layer in the composites developed. At lower load application, the oxide film formed appeared to be at the formative stage, this enhances the wear rate formation. A similar trend was reported from the work of Radhika et al.^14^. However, as the load increases, more particles are being pulled out of the reinforcement, thereby leading to plastic deformation. Previous studies by Uthayakumar et al.^15^ reported that particle pulled out of the reinforcement could form a third body abrasion condition.

### Specific wear rate

**Figure 7:**
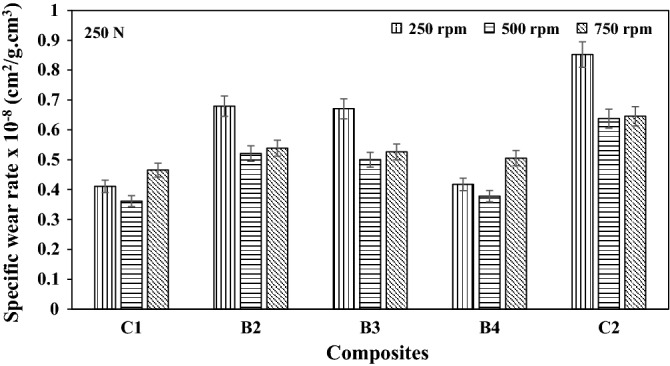
Specific wear rate of composites at load 250 N load under different speed.

**Figure 8:**
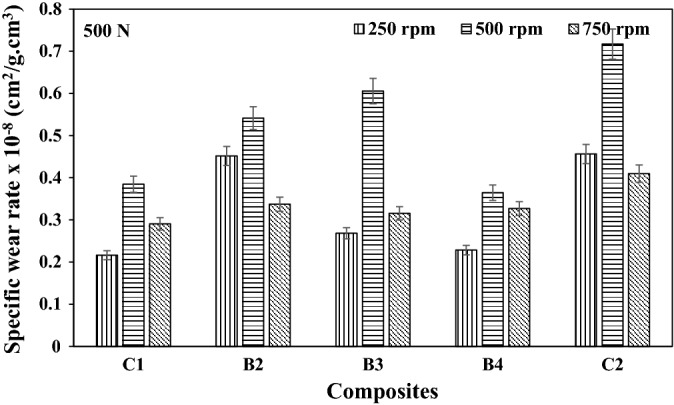
Specific wear rate of composites at load 500 N load under different speed.

**Figure 9:**
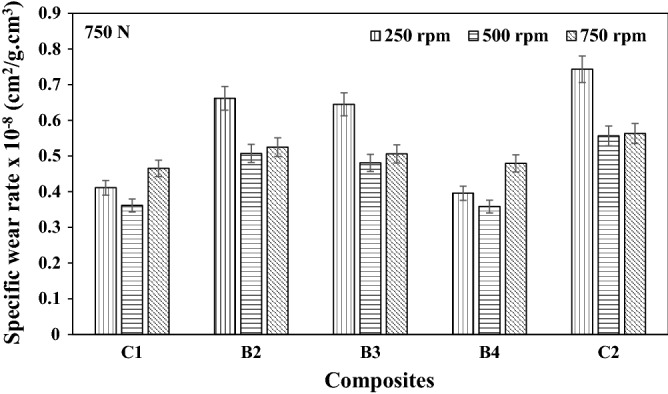
Specific wear rate of composites at load 750 N load under different speed.

The specific wear rate of the composites at different load applications and speeds are as presented in Figs. 7, 8, and 9 respectively. The specific wear rate (cm^2^/g.cm^3^) is a measure of the total area or amount of materials that are cut off from the surface of the hybrid composite by one Newton load/force^16^. It could be observed from Fig. 9, that the lowest specific wear rate of 3.587 × 10^–9^ cm^2^/g.cm^3^ was obtained by B4 hybrid composite at the maximum wear load of 750N and sliding speed of 500 rpm. Increased specific wear rate as observed for the C2 composite having a single reinforcement content of 10wt.% SBRC-BLA could be attributed to catastrophic ploughing and dislodging of the revealed reinforcement particles from the topmost surface of the composites as soon as they come in contact with the progressively sliding abrasive disc^16^.

### Statistical analysis, modelling and optimization of wear properties and parameters

The design summary, showing the build information for the factor and response variables are presented in Tables 3, 4 respectively.

**Table 3:** Build information for the design factors.

Factor	Name	Units	Type	Minimum	Maximum	Coded Low	Coded High	Mean	Std. Dev
A	Speed	rpm	Numeric	417.11	750.00	− 1 ↔ 250.00	+ 1 ↔ 750.00	583.56	137.44
B	Load	N	Numeric	417.11	750.00	− 1 ↔ 250.00	+ 1 ↔ 750.00	583.56	137.44
C	Composition	%.wt	Categoric	C1	C2			**Levels:**	5

**Table 4:** Design build information for the response variables.

Response	Name	Units	Obs	Analysis	Mini	Maxi	Mean	Std. Dev	Ratio	Transform	Model
R1	Wear rate	mm^2^/min	45	Polynomial	0.611	2.100	1.31	0.37	3.44	None	Quadratic
R2	SWR	cm^2^/g·cm^3^	45	Polynomial	0.216	0.852	0.48	0.14	3.94	None	Quadratic
R3	Wear loss	g	45	Polynomial	0.017	0.433	0.21	0.11	25.47	None	Quadratic

#### Analysis of variance (ANOVA) and fits statistics

The validity of the developed predictive models is tested statistically using the analysis of variance technique (ANOVA). ANOVA is useful for establishing the statistical adequacy, significance and practical usefulness of the various factors and the response variables. ANOVA also ascertain the impacts of the experimental wear parameters and filler contents on the wear performance of the studied composites. The relationship between the factors and the dependent response variables (wear properties) in improving the properties of the developed metal matrix composites was established. The results of ANOVA and summary of fit statistics are presented in Tables 5, 6, and 7. From the Tables, it could be observed that there is a significant effect between the factors and response variables based on a 95% confidence level. The P-values (probability values) obtained for all the response variables for each of the factor were less than 0.0500 which is an indication that the developed model terms are significant. Values greater than 0.1000 indicate the model terms that are not significant. The F-values presented for all the response variables are also significant, and is a good sign that the input parameters has a positive impact on the responses^17,18^. The fitness of the designed model with respect to their response output is represented by Adjusted R^2^, Predicted R^2^, and Adeq precision. For accuracy to be obtained from a design model, the difference between the adjusted R^2^ and predicted R^2^ value will be less than 0.2^19,20^, hence, it could be seen that a reasonable agreement existed in the responses analyzed in this work. An adequate signal to noise is obtained for all the response output. According to statistical rule, a desirable Adeq precision ratio should be greater than 4 for an adequate signal to be obtained^19,21,22^. Therefore, the ratio of the analyzed response variables is seen to be greater than 4, which clearly indicate that the developed models are adequate to navigate the design space.

**Table 5:** ANOVA and summary of fit statistics for response 1 (wear rate).

Source	Sum of squares	df	Mean square	F value	*p* value	
Model	4.39	17	0.2585	4.29	0.0004	Significant
A-Speed	0.0880	1	0.0880	1.46	0.2372	
B-Load	0.0000	1	0.0000	0.0000	1.0000	
C-Composition	2.24	4	0.5611	9.32	< 0.0001	
AB	0.0000	1	0.0000	0.0000	1.0000	
AC	0.5299	4	0.1325	2.20	0.0958	
BC	0.0000	4	0.0000	0.0000	1.0000	
A^2^	0.0005	1	0.0005	0.0090	0.9252	
B^2^	1.53	1	1.53	25.41	< 0.0001	
Residual	1.63	27	0.0602			
Cor total	6.02	44				
SD	0.2454		R^2^	0.7299		
Mean	1.31		Adjusted R^2^	0.5598		
C.V. %	18.70		Predicted R^2^	0.3961		
			Adeq Precision	8.0991

**Table 6:** ANOVA and summary of fit statistics for response 2 (specific wear rate).

Source	Sum of squares	df	Mean square	F value	*p* value	
Model	0.6581	17	0.0387	4.63	0.0002	Significant
A-Speed	0.0123	1	0.0123	1.47	0.2353	
B-Load	0.0067	1	0.0067	0.8004	0.3789	
C-Composition	0.3867	4	0.0967	11.56	< 0.0001	
AB	0.0000	1	0.0000	0.0057	0.9403	
AC	0.0716	4	0.0179	2.14	0.1033	
BC	0.0074	4	0.0019	0.2219	0.9239	
A^2^	0.0002	1	0.0002	0.0268	0.8712	
B^2^	0.1730	1	0.1730	20.69	0.0001	
Residual	0.2258	27	0.0084			
Cor total	0.8839	44				
Std. Dev	0.0915		R^2^	0.7445		
Mean	0.4819		Adjusted R^2^	0.5837		
C.V. %	18.98		Predicted R^2^	0.4324		
			Adeq precision	9.1965

**Table 7:** ANOVA and summary of fit statistics for response 3 (wear loss).

Source	Sum of squares	df	Mean square	F value	*p* value	Source
Model	0.4661	17	0.0274	12.57	< 0.0001	Significant
A-Speed	0.2980	1	0.2980	136.65	< 0.0001	
B-Load	0.0974	1	0.0974	44.64	< 0.0001	
C-Composition	0.0310	4	0.0078	3.56	0.0186	
AB	0.0095	1	0.0095	4.34	0.0469	
AC	0.0008	4	0.0002	0.0940	0.9835	
BC	0.0002	4	0.0000	0.0221	0.9990	
A^2^	0.0282	1	0.0282	12.91	0.0013	
B^2^	0.0011	1	0.0011	0.4992	0.4859	
Residual	0.0589	27	0.0022			
Cor total	0.5250	44				
Std. Dev	0.0467		R^2^	0.8878		
Mean	0.2070		Adjusted R^2^	0.8172		
C.V. %	22.56		Predicted R^2^	0.6964		
			Adeq precision	12.7834		

#### Graphical analysis of model

**Figure 10:**
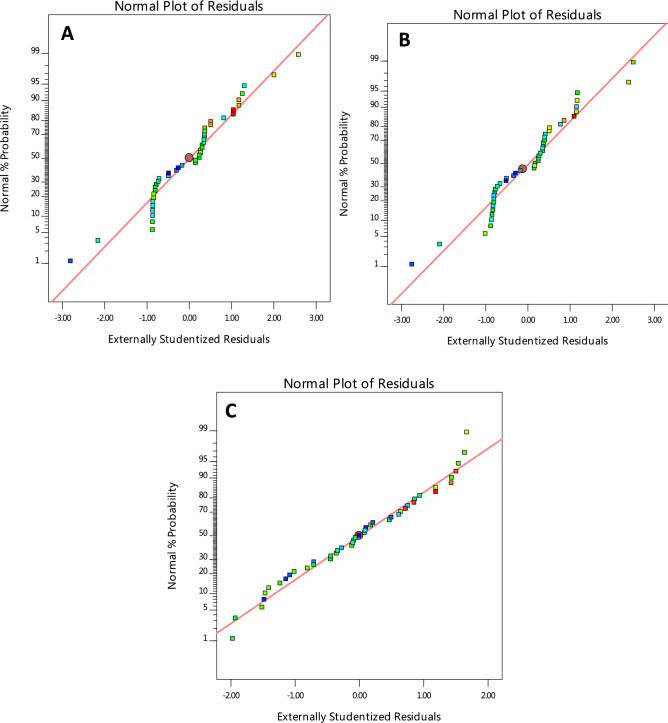
Normal probability plots of residuals for (**A**) wear rate, (**B**) specific wear rate, & (**C**) wear loss.

**Figure 11:**
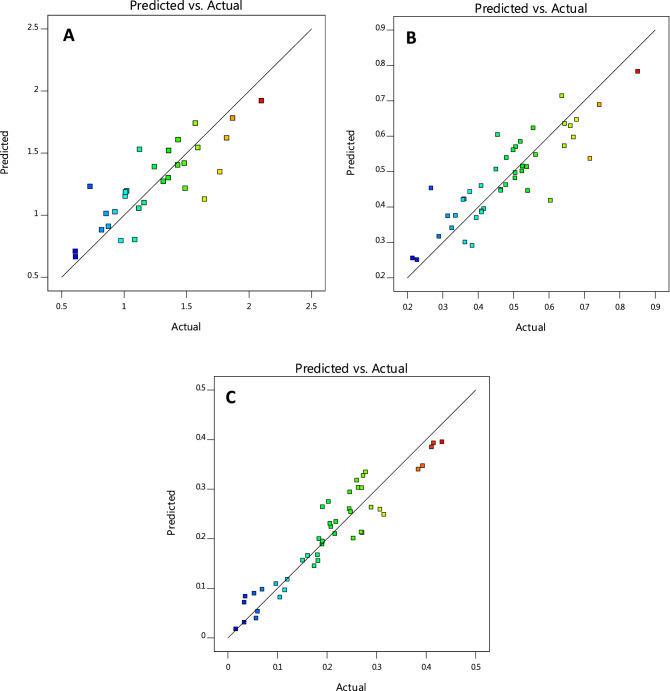
Model graphs of predicted versus actual design values for (**A**) wear rate, (**B**) specific wear rate, & (**C**) wear loss.

**Figure 12:**
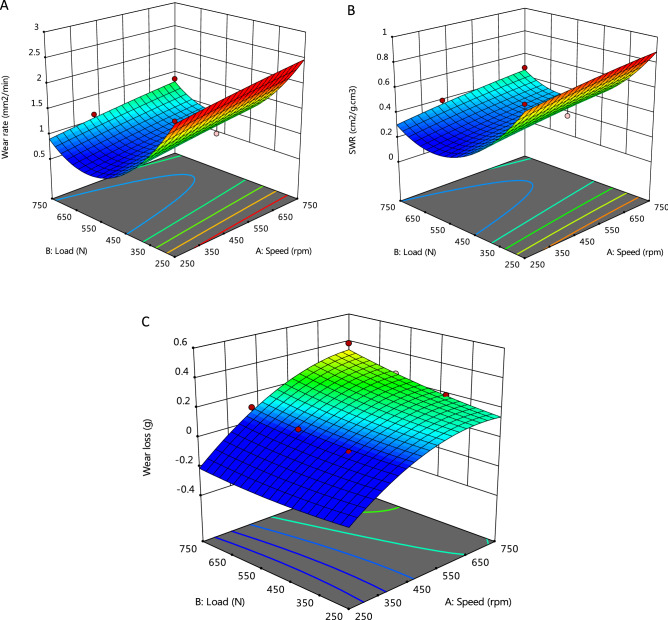
Interactive 3D surface model graphs for (**A**) wear rate, (**B**) specific wear rate, & (**C**) wear loss, against the design factors.

**Figure 13:**
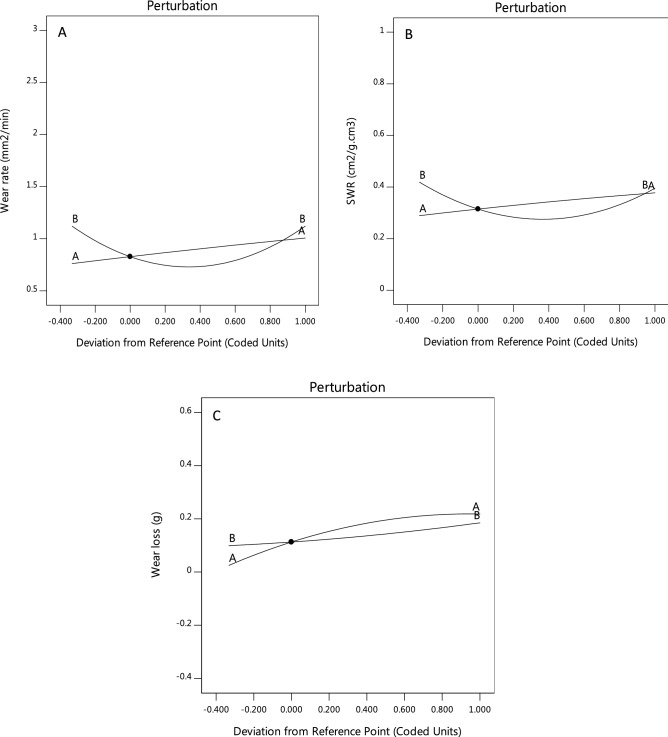
Perturbation plots for (**A**) wear rate, (**B**) specific wear rate, & (**C**) wear loss.

Design-Expert software provides various graphs to help interpret the selected fit model. Diagnostic plots and model graphs were used to analyze and interpret the fit models. Normal probability plots (Figs. 10A-C) show the linear effect of changing the level of one factor. The normal probability plots are designed to indicate whether the residuals follow a normal distribution, thus follow the straight line. Though, some scatter may be expected even with normal data. Definite patterns, like an “S-shaped” curve are clear indication that a transformation of the response may provide a better analysis. In this case, all the responses exhibit a linear distribution. The predicted versus actual model graphs of Figs. 11A-C show the actual observed response values versus the predicted response values and detect observations that are not easily predicted by the model. It could be observed that the data points are split evenly by the 45-degree line, indicating that the design models are sufficient to predict the responses^16,23^. For response surface designs, the primary model graphs are the 3D and contour plots. The contour plot is a two-dimensional (2D) representation of a response variable plotted against combinations of numeric factors. It can show the relationship between the responses and the numeric factors^24^. The 3D Surface plot is a projection of the contour plot giving shape in addition to the color and contour. Figure 12A–C are interactive 3D surface plots, showing the degree of interaction among the factors and the response variables at the specified categoric factor level (B4). An interaction occurs when the response is different depending on the settings of the two numeric factors (Load and speed)^25^. They will appear with two non-parallel lines, indicating that the effect of one factor depends on the level of the other. The perturbation plot of Fig. 13A–C is employed to compare the effects of all the factors at a particular point in the design space. The response is plotted by changing only one factor over its range while holding all the other factors constant. By default, Design-Expert sets the reference point at the midpoint (coded 0) of all the factors. This can be changed to be any point (perhaps the optimal run conditions) by using the Factors Tool. A steep slope or curvature in a factor shows that the response is sensitive to that factor. A relatively flat line shows insensitivity to change in that particular factor^18,20^. If there are two or more factors, the perturbation plot could be used to find those factors that most affect the response. From the perturbation curves (Fig. 13A–C), it could be stated that the variation in speed does not have marked impact on the wear rate and specific wear rate, but does on the wear loss. Axes on the contour plots are chosen based on these influential factors.

#### Parametric optimization solution

**Figure 14:**
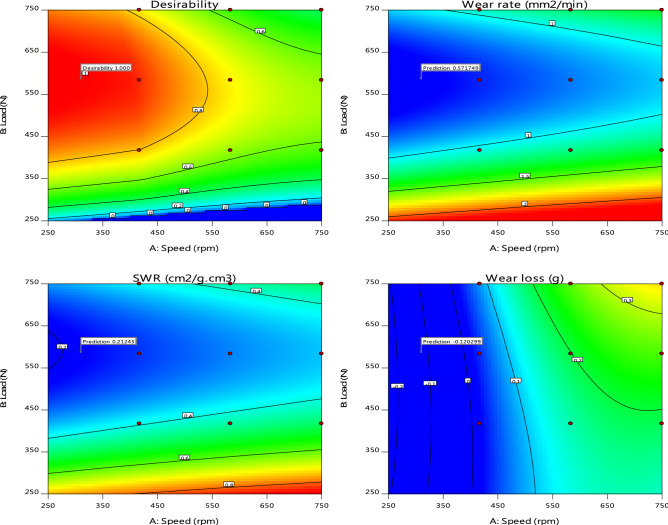
Contour plots of the numerical optimization results.

**Figure 15:**
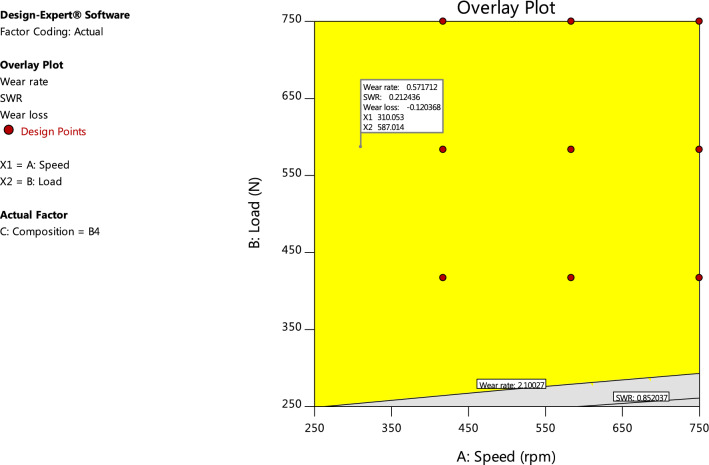
Overlay plot showing the numerical optimization solution.

Numerical Optimization criteria is set to optimize any combination of one or more goals. The goals may apply to either factor or response variables. The possible goals normally set during numerical optimization are: maximize, minimize, target, within range, none (for responses only) and set to an exact value (factors only). A minimum and a maximum level (lower and upper limits) must be provided for each parameter included in the optimization. The “importance” of a goal can be changed in relation to the other goals. The default is for all goals to be equally important at a setting of 3 pluses (+ + +). Table 8 summarizes the criteria constraints applied to find the optimal settings and solutions for the numerical optimization process. From the numerical optimization report, one solution that best meet the specified criteria is chosen as the optimal solution. This is most times the solution that have the highest desirability scores, indicating there is an island of acceptable outcomes. The optimal solution is presented graphically using contour and overlay plots (Figs. 14, 15). The overlay graph produces a single plot highlighting the “sweet spot”, where all response criteria can be met. It is also used to show the limits of failure in a process^17,18,20^. The contours are plotted at the limits specified by the criteria. Bright yellow colour by default defines the acceptable factor settings, while grey colour by default identifies the unacceptable factor settings. The selected numerical optimization solutions are carried over and displayed on the contour and overlay plots as flags if the graph is on the correct slice. The results of numerical optimization indicate that at a load, speed and hybrid filler composition level of 587.014N, 310.053 rpm and B_4_(4%SBRC-BL + 6% alumina) respectively, optimal responses in wear rate (0.572mm^2^/min), specific wear rate (0.212cm^2^/g.cm^3^) and wear loss (0.120 g) would be obtained for the developed AA6063 based composite.

**Table 8:** Numerical optimization constraints and target goals.

Name	Goal	Lower limit	Upper limit	Importance
A:Speed	In range	250	750	3
B:Load	In range	250	750	3
C:Composition	In range	C1	C2	3
Wear rate	Minimize	0.610875	2.10027	3
SWR	Minimize	0.216239	0.852037	3
Wear loss	Minimize	0.017	0.433	3

## Conclusion

Hybrid composites of AA6063 reinforced with varying weight concentrations of Al_2_O_3_ and SBRC from bamboo leaf ash was successively developed by double stir casting technique. The hybrid composites reinforced with 6% alumina + 4%SBRC from BLA, designated as B4 exhibited better abrasive wear properties for all the considered wear parameters. The application of statistical design model based on central composite design type of RSM to simulate wear experiments is effective and adequately predict the abrasive wear response of the hybrid reinforced aluminium 6063 composites under the considered test conditions (varying load, speed and hybrid filler weight compositions). The optimally predicted and experimental values of wear rate are in good agreement, as both best experimental and predicted optimal wear properties were obtained by B4 weight ratio of the hybrid reinforcements at all imposed wear parameters (load and speed), validating the remarkable capability of the chosen design model type in relation to the present investigation.

## Data Availability

All data generated or analyzed during this study are included in this published article.
